# A Mixed Methods Study Evaluating Public Understanding of “Plastic” and “Cosmetic” Surgery

**DOI:** 10.1093/asjof/ojae007.048

**Published:** 2024-04-12

**Authors:** Shirley Chen, Harrison Thomas, Benjamin Park, Alan Makhoul, Galen Perdikis, Catherine Hammack-Aviran, Brian Drolet

**Affiliations:** Vanderbilt University Medical Center, Nashville, TN; Vanderbilt University School of Medicine, Nashville, TN; Vanderbilt University Medical Center, Nashville, TN; University of Pennsylvania, Philadelphia, PA; Vanderbilt University Medical Center, Nashville, TN; Belmont University, Nashville, TN; Vanderbilt University Medical Center, Nashville, TN

## Abstract

**Goals/Purpose:**

The aesthetic marketplace has expanded significantly over the past decade. There has been a corresponding expansion in the types of providers offering aesthetic procedures and the organizations certifying these providers. These include the American Board of Plastic Surgery (ABPS) and the American Board of Cosmetic Surgery (ABCS). Because patients self-pay for aesthetic procedures, they exercise a high degree of autonomy when choosing a provider. Given the need to support patients in making informed decisions, this study aimed to investigate how the public understands the distinction between “plastic” and “cosmetic” surgery using a mixed methods approach.

**Methods/Technique:**

The study was completed in two parts: qualitative interviews and a survey.

Semi-structured qualitative interviews were conducted with post-operative aesthetic surgery patients. An interview guide asking about participants’ understanding of “plastic” versus “cosmetic” surgery was developed in collaboration with content and methodology experts, then refined through pilot testing. Purposive sampling of patients maximized representation of demographic characteristics and type of surgery. A codebook was constructed using constant comparative grounded theory, which was then applied to interview transcripts to identify emergent themes.

Next, a survey instrument investigating the public’s perception of “plastic” versus “cosmetic” surgery and ABPS versus ABCS was formulated from themes identified in the interviews. Pre-testing by cognitive interviews confirmed item validity, and pilot testing confirmed internal consistency. The survey was distributed on Mechanical Turk, with respondents receiving $1.50 as compensation.

**Results/Complications:**

To achieve thematic saturation, 24 patients were interviewed. Intercoder reliability was high (κ > 0.8). Most (18/24) participants viewed plastic and cosmetic surgery as the same (Table 1). Almost all (23/24) discussed the aesthetic component of plastic surgery while fewer (16/24) participants mentioned the aesthetic component of cosmetic surgery (Table 1). Furthermore, some participants (7/24) misunderstood the scope of cosmetic surgery, including reconstructive procedures under its umbrella (Table 1).

600 respondents completed the survey. When asked how the scopes of plastic and cosmetic surgery relate to each other, a plurality of respondents believed plastic and cosmetic surgery overlap, but each has unique features (35.4%, Figure 1). 25.9% thought plastic and cosmetic surgery were the same, and only 13.9% correctly selected that plastic surgery is inclusive of all cosmetic surgery (Figure 1). When asked who they would go see for various procedures, a plurality of respondents chose a cosmetic surgeon (44.7-49.0% depending on the procedure, Figure 2). A minority stated they would see a plastic surgeon (23.4-29.9% depending on the procedure, Figure 2) or had no preference between a plastic surgeon and a cosmetic surgeon (23.4-28.8% depending on the procedure, Figure 2). However, when asked who they thought was more qualified to perform aesthetic surgery, a plurality of respondents selected an ABPS-certified physician (ABPS 42.2%, ABCS 25.9%, both equally 26.8%, Figure 3).

**Conclusion:**

These results demonstrate that despite efforts by professional plastic surgery societies, confusion remains about the difference between “plastic” and “cosmetic” surgery, with a portion of the public conflating the two. Furthermore, the survey results indicate that although the public recognizes ABPS certification as a signifier of legitimacy, they may view these physicians as “cosmetic surgeons”. These gaps in public knowledge demonstrate the need for more robust public education campaigns and legislative initiatives to increase transparency and aid patients in making informed choices about their aesthetic providers.

